# Classification differentiates clinical and neuroanatomic features of cerebral small vessel disease

**DOI:** 10.1093/braincomms/fcab107

**Published:** 2021-05-20

**Authors:** Kun-Hsien Chou, Pei-Lin Lee, Li-Ning Peng, Wei-Ju Lee, Pei-Ning Wang, Liang-Kung Chen, Ching-Po Lin, Chih-Ping Chung

**Affiliations:** 1 Institute of Neuroscience, National Yang Ming Chiao Tung University College of Medicine, Taipei 112, Taiwan; 2 Brain Research Center, National Yang Ming Chiao Tung University College of Medicine, Taipei 112, Taiwan; 3 Department of Neurology in School of Medicine, National Yang Ming Chiao Tung University College of Medicine, Taipei 112, Taiwan; 4 Aging and Health Research Center, National Yang Ming Chiao Tung University College of Medicine, Taipei 112, Taiwan; 5 Center for Geriatric and Gerontology, Taipei Veterans General Hospital, Taipei 112, Taiwan; 6 Department of Family Medicine, Taipei Veterans General Hospital Yuanshan Branch, Yi-Lan 264, Taiwan; 7 Department of Neurology, Neurological Institute, Taipei Veterans General Hospital, Taipei 112, Taiwan

**Keywords:** cerebral small vessel disease, stratification, grey-matter volume, white-matter microstructure integrity

## Abstract

Age-related cerebral small vessel disease involves heterogeneous pathogenesis, such as arteriosclerosis/lipohyalinosis and cerebral amyloid angiopathy. MRI can visualize the brain lesions attributable to small vessel disease pathologies, including white-matter hyperintensities, lacune and cerebral microbleeds. However, these MRI markers usually coexist in small vessel disease of different aetiologies. Currently, there is no available classification integrating these neuroimaging markers for differentiating clinical and neuroanatomic features of small vessel disease yet. In this study, we tested whether our proposed stratification scheme could characterize specific clinical, neuroanatomic and potentially pathogenesis/aetiologies in classified small vessel disease subtypes. Cross-sectional analyses from a community-based non-demented non-stroke cohort consisting of ≥50 years old individuals were conducted. All participants were scanned 3T brain MRI for small vessel disease detection and neuroanatomic measurements and underwent physical and cognitive assessments. Study population were classified into robust and four small vessel disease groups based on imaging markers indicating (i) bleeding or non-bleeding; (ii) specific location of cerebral microbleeds; and (iii) the severity and combination of white-matter hyperintensities and lacune. We used whole-brain voxel-based morphometry analyses and tract-based spatial statistics to evaluate the regional grey-matter volume and white-matter microstructure integrity for comparisons among groups. Among the 735 participants with eligible brain MRI images, quality screening qualified 670 for grey-matter volume analyses and 617 for white-matter microstructural analyses. Common and distinct patterns of the clinical and neuroimaging manifestations were found in the stratified four small vessel disease subgroups. Hierarchical clustering analysis revealed that small vessel disease type 4 had features distinct from the small vessel disease types 1, 2 and 3. Abnormal white-matter microstructures and cognitive function but preserved physical function and grey-matter volume were found in small vessel disease type 4. Among small vessel disease types 1, 2 and 3, there were similar characteristics but different severity; the clinical features showed both physical frail and cognitive impairment and the neuroanatomic features revealed frontal–subcortical white-matter microstructures and remote, diffuse cortical abnormalities. This novel stratification scheme highlights the distinct clinical and neuroanatomic features of small vessel disease and the possible underlying pathogenesis. It could have potential application in research and clinical settings.

## Graphical Abstract

**Graphical Abstract:**
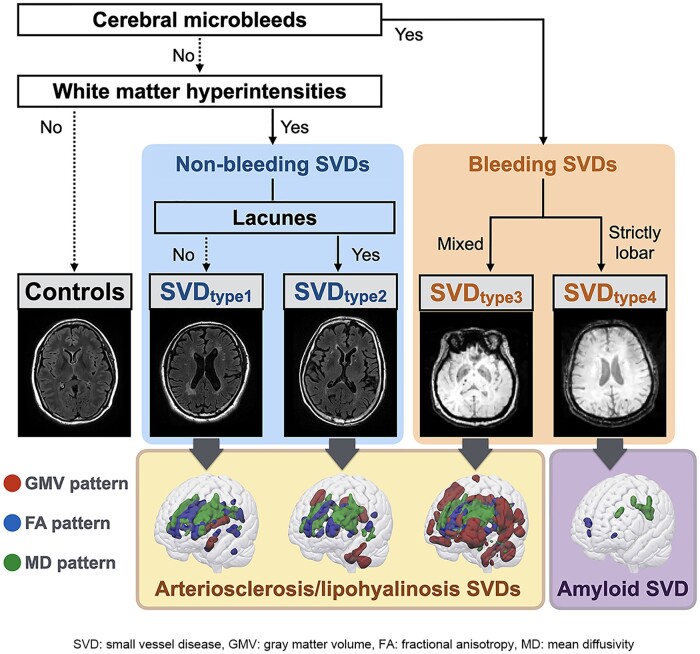

Abbreviated summary

Chou et al. propose a novel classification for cerebral small vessel disease and report the common and distinct patterns of the clinical and neuroimaging manifestations in the stratified four subgroups. It could be used in clinical and research settings to differentiate arteriosclerosis and cerebral amyloid angiopathy and also their severity.

## Introduction

Cerebral small vessel disease (SVD) adversely affects cognitive and physical function and is an important cause of age-related disability.^1^^,^^2^ The aetiologies of SVD are heterogeneous and include the two most recognized ones, arteriosclerosis/lipohyalinosis and amyloid accumulation [cerebral amyloid angiopathy (CAA)], in brain microvessels.^1–3^ Owing to developments in MRI, we can now identify the brain lesions attributable to SVD at an earlier stage.^1^^,^^4^ These MRI markers of SVD are also heterogeneous and include ischaemic lesions such as white-matter hyperintensities (WMH) and lacune(s), and haemorrhagic lesions, the cerebral microbleeds (CMB).^1^^,^^5^ These lesions are closely related and usually co-occur in different SVD aetiologies.^1^^,^^5^ Current SVD indices are often derived by summing the different MRI markers that are present in an individual.^6^^,^^7^ These scoring methods may reflect the SVD burden and predict prognosis.^6–8^ However, they do not provide information about the underlying pathogenesis of SVD.

Here, we propose a novel stratification scheme of SVD using three common MRI markers, WMH, lacune and CMB, based on (i) bleeding or non-bleeding; (ii) CMB locations that can differentiate CAA from arteriosclerosis/lipohyalinosis microvasculopathy^9^^,^^10^; and (iii) the severity and combination of WMH and lacune. We investigated whether this classification could differentiate clinical and neuroanatomic alterations, and potentially the pathophysiology, of SVD.

## Materials and methods

### Subjects

Participants were from the I-Lan Longitudinal Aging Study (ILAS) cohort, which is a community-based ageing cohort study in the I-Lan County of Taiwan that aims to evaluate the mechanisms of ageing.^11–13^ Community-dwelling adults aged ≥50 years from Yuanshan Township in I-Lan County were invited to participate. The inclusion criteria of the ILAS were as follows: (i) inhabitants of I-Lan County who were not planning to move soon and (ii) aged ≥50 years. Subjects that met any of the following conditions were excluded: (i) inability to communicate and complete an interview; (ii) inability to complete a simple motor task (e.g. a 6-m walk) due to functional disability; (iii) presence of any major illness with associated decreased life expectancy (less than six months); (4) presence of any contraindication for MRI (such as metal implants); and (5) institutionalisation for any reason. In addition, subjects diagnosed with neuropsychiatric diseases, such as dementia, stroke, brain tumour or major depression, were excluded in the present study. The present study used demographic and brain MRI data from the initial sampling wave of the ILAS (recruited between January 2011 and July 2014). The study was approved by the Institutional Review Board of the National Yang Ming University, Taipei, Taiwan. All participants gave their written informed consent.

### Assessment of physical frailty

Physical frailty was assessed according to the Cardiovascular Health Study (CHS) criteria.^14^^,^^15^ The ﬁve components of the CHS frailty criteria are weight loss, poor endurance and energy (exhaustion), weakness, slowness and low physical activity level. The severity of the frail status was rated as a CHS score from 0 to 5. A person was considered frail if three or more components were present. The presence of weight loss was identified as unintentional weight loss of >5% in the last year. Exhaustion was determined using two CES-D statements. Weakness was defined by the results of low handgrip strength (measured using digital dynamometry, Smedley’s Dynamometer; TTM, Tokyo, Japan), and slowness was defined by the results of the slow walking speed (6-m walking test). Physical activity was assessed by the energy consumption of different types of physical activities, according to the criteria of the International Physical Activity Questionnaire-Taiwan edition.^16^ Weakness, slowness and low physical activity referred to performances that were lower than the sex-specific lowest 20% of the ILAS population (for males and females: International Physical Activity Questionnaire score ≤ 7497 and 6930; walking speed ≤ 1.20 and 1.10 m/s; handgrip strength ≤ 28.00 and 17.00 kg, respectively).

### Assessment of cognitive function

All participants received a face-to-face neuropsychological assessment administered by trained interviewers. We used the Mini-Mental State Examination (MMSE) to evaluate global cognitive performance. Global cognitive impairment was defined as an MMSE score <24 in well-educated subjects (education year ≥ 6 years) or <14 in less educated subjects (education year < 6 years).^17^^,^^18^ This criterion was derived from a field survey in Taiwan, which included 2753 males and 2544 females from four urban and four rural communities.^17^^,^^18^

We also performed neuropsychological tests across multiple domains in each participant:

- Verbal memory: 10 min delayed recall in the Chinese Version Verbal Learning Test.^19^
- Visuospatial function: Taylor Complex Figure Test.^18^
- Executive function: Clock Drawing Test.^20^

### Brain MRI acquisition

Multimodal neuroimaging acquisition was performed at the National Yang Ming University to obtain six image-based markers for each participant, including grey-matter volume (GMV), WMH, lacune(s), CMB, fractional anisotropy (FA) and mean diffusivity (MD). All MRI scans were collected on a single 3 T Siemens MRI (Siemens Magnetom Tim Trio, Erlangen, Germany) with a vendor-supplied 12-channel phased-array head coil. All acquired whole-brain MRI scans were without inter-slice gap and interpolation. The following imaging sequences were used. First, T_1_-weighted images were acquired using a three-dimensional T_1_-weighted magnetisation-prepared rapid-acquisition gradient echo sequence [repetition time (TR)/echo time (TE)/inversion time (TI) = 3500/3.5/1100 ms; flip angle = 7°; number of excitations (NEX) = 1; field of view (FOV) = 256 × 256 mm; matrix size = 256 × 256; 192 sagittal slices; and voxel size = 1.0 mm^3^]. Second, T_2_-weighted fluid-attenuated inversion recovery (FLAIR) images were acquired using a two-dimensional T_2_-weighted FLAIR multishot turbo-spin-echo sequence (TR/TE/TI = 9000/143/2500 ms; flip angle = 130°; NEX = 1; FOV = 220 × 220 mm; matrix size = 320 × 320, echo train length = 35; 63 axial slices; and voxel size = 0.69 mm × 0.69 mm × 2.0 mm). Third, susceptibility-weighted images (SWI) were acquired using a three-dimensional SWI sequence (TR/TE = 28/21 ms; flip angle = 15°, FOV = 256 × 224 mm; matrix size = 256 × 224; 88 axial slices; bandwidth =120 Hz/Px; and voxel size = 1.0 × 1.0 × 2.0 mm). Finally, diffusion MRI data were acquired using a single-shot spin-echo echo-planar imaging sequence (TR/TE = 11 000/104 ms; flip angle = 90°; NEX = 3; FOV = 256 × 256 mm; matrix size = 128 × 128; 70 axial slices; 30 non-collinear diffusion gradient directions with b value of 1000 s/mm^2^; 3 b0 images; and voxel size = 2.0 × 2.0 × 2.0 mm). Before the image pre-processing, all the acquired MRI scans were visually examined by an experienced neuroradiologist to exclude any data with severe motion artefacts or gross brain abnormalities, including trauma, tumour, and intracerebral haemorrhagic or territorial infarct lesions (in the territory of large arteries or their branches but not of a perforating artery).

### Detection and assessment of MRI SVD markers

CMBs were defined as small, rounded or circular, well-defined, hypointense lesions, within the brain parenchyma, with clear margins and ≤10 mm in size on SWI.^21^^,^^22^ Microbleed mimics, such as vessels, calcification, partial volume, air-bone interfaces and haemorrhage within or adjacent to an infarct, were carefully excluded. We used the microbleed anatomical rating scale to measure the presence, amount and topographic distribution of CMBs.^22^ Intra-rater reliability was assessed by evaluating CMBs in 20 randomly sampled images at a separate time (*K* = 0.83; 95% confidence interval, 0.79–0.90). We also re-assessed CMBs in 25 randomly sampled images previously assessed by Dr Chung and another investigator (*K* = 0.82; 95% confidence interval, 0.79–0.88). CMBs were classified into deep, infratentorial and lobar categories. Lobar topography was determined according to Stark and Bradley^23^ and included cortical and subcortical regions including subcortical U fibres. Lobar CMBs were assessed in the frontal, parietal, temporal and occipital regions. Deep regions included the basal ganglia, thalamus, internal capsule, external capsule, corpus callosum and deep/periventricular WM, whereas infratentorial regions included the brainstem and cerebellum. Subjects with CMBs were divided into two types according to the CMB topography: strictly lobar (CMB exclusively located in lobar regions) and mixed CMB (deep or/and infratentorial CMB with or without lobar CMB). Lacune were assessed using T_2_-weighted FLAIR anatomical scans. Lacunes are defined as small (<15 mm in diameter) CSF-containing cavities, located in the deep grey or white matter, with adjacent WMH.^1^

### Proposed scheme for SVD stratification

**Figure 1:**
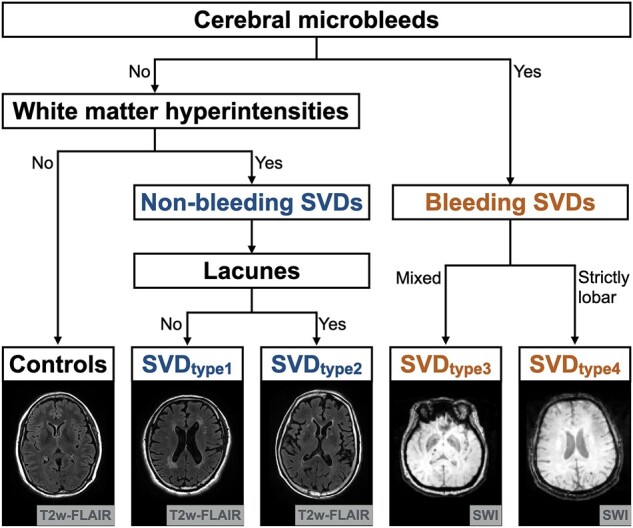
**Methodological sequence for phenotyping age-related cerebral small vessel disease**. The stratification scheme had three steps in order: whether there was (i) presence of cerebral microbleeds; (ii) presence of severe WMH (defined as >50th percentile of WMH/TIV ratio); and (iii) a combination of lacune with severe WMH or the geographic patterns of cerebral microbleeds (mixed or strictly lobar) if cerebral microbleeds were present. People without cerebral microbleeds and severe WMH were classified as the control group. SVD, cerebral small vessel disease; TIV, total intracranial volume; WMH, white-matter hyperintensities.

The stratification scheme had three steps in order: whether there was (i) presence of CMB; (ii) presence of severe WMH [defined as >50th percentile of WMH/total intracranial volume (TIV) ratio (0.07%)]; and (iii) a combination of lacune with severe WMH or certain geographic pattern of CMB (mixed or strictly lobar) when CMB was present (Fig. 1). People without CMB and severe WMH were allocated to the control (robust) group. There were two types of non-bleeding SVD (WMH without or with lacune; SVD type 1 and 2) and two types of bleeding SVD (mixed or strictly lobar CMB; SVD type 3 and 4).

### Volume quantification of global and voxel-wise tissue and WMH

We used the previously proposed analytical framework to extract both global-wise tissue and WMH volume as well as voxel-wise GMV information for each patient.^13^^,^^24^ The following analyses were conducted with Statistical Parametric Mapping (SPM12, version 7487, Wellcome Institute of Neurology, University College London, UK, http://www.fil.ion.ucl.ac.uk/spm/) and Matlab R2016a (The Mathworks, Inc., Natick, MA, USA) using default settings. A brief description of the analytical procedure is as follows: (i) using the Lesion Segmentation Toolbox (LST, version 3.0.0, https://www.applied-statistics.de/lst.html),^25^ individual T_2_-weighted FLAIR scans were first affine-registered to the corresponding T_1_-weighted scan. They then served as the inputs for generating a native T_1_ space WMH probability map and lesion-filled T_1_ image; (ii) to further minimise the registration error of the following spatial normalisation procedure, a centre-of-mass approach was applied to reorient individual lesion-filled T_1_-weighted scans automatically; (iii) using SPM12’s ‘Segment’ module, these native-space lesion-filled T_1_-weighted scans were corrected for intensity nonuniformities, segmented to produce GM, WM and CSF tissue probability maps and then rigid-aligned to the Montreal Neurological Institute (MNI) space. To further improve the anatomical plausibility of the subcortical brain structure tissue segmentation, we replaced the original SPM12 tissue a priori with the newly enhanced tissue probability maps using the above tissue segmentation procedure^26^; (iv) using the Diffeomorphic Anatomical Registration Exponentiated Lie Algebra approach,^27^ the study-specific GM and WM templates were generated based on all rigid-aligned GM and WM segments of study participants, and the flow field of each participant was also calculated; (v) to perform the voxel-wise statistical analyses of GMV in the standard MNI space, the additional affine transformation was estimated from the group-specific DARTEL GM template and the a priori MNI space GM probability map. Combined with the previously obtained flow fields, the individual native T_1_ space WMH, GM and WM probability maps could be spatially transformed into the MNI space and further modulated with the corresponding Jacobian determinants to obtain the actual tissue volumetric information; (vi) the global tissue volume, including GM, WM, CSF, WMH and TIV was calculated in the MNI space; and (vii) the resultant modulated GMV maps were smoothed with an 8-mm full-width at half-maximum isotropic Gaussian kernel. Voxels were further excluded with a GM probability lower than 0.2. All pre-processed data after each analytical step were visually checked to rule out data with inaccurate pre-processing results. Finally, using the individual modulated GMV map as inputs, the ‘Check Sample Homogeneity’ function, which is available in the Computational Anatomy Toolbox (CAT12, version 1653, http://www. neuro.uni-jena.de/cat/) was also applied to evaluate the sample homogeneity for quality control. Individual data with an overall covariance between two standard deviations were additionally checked. No subjects were excluded from this quality control step. These smoothed GMV maps were entered as inputs for the subsequent voxel-wise statistical analyses.

### WM microstructure integrity quantification

Diffusion MRI data were all processed using FSL version 5.0.9 (Functional Magnetic Resonance Imaging of the Brain Software Library; http://www.fmrib.ox.uk/fsl). All diffusion MRI data underwent a careful quality check for head motion and scanner instability-induced artefacts. To further reduce image distortion, which originates from eddy current and simple subject head motion, the individual diffusion-weighted images were affine-registered to the corresponding non-diffusion-weighted images using the eddy_correct tool (part of FSL). Voxel-wise statistical analyses of the FA and MD data were carried out using tract-based spatial statistics (TBSS)^28^ and the following five steps: (i) individual voxel-wise FA and MD maps were calculated by fitting a tensor model to motion-corrected diffusion MRI data using FMRIB’s diffusion toolbox (part of FSL); (ii) to remove non-brain tissue and background noise, the brain extraction tool (part of FSL)^29^ was applied to the non-diffusion-weighted image and the resultant brain mask was further applied to the FA and MD maps for each individual; (iii) using FMRIB’s nonlinear image tool (part of FSL), all participants’ FA maps were nonlinearly aligned to a pre-defined 1 × 1 × 1 mm FMRIB58_FA standard MNI space template and then averaged to generate a study-specific mean FA image; (iv) this study-specific mean FA image was subsequently thinned to create a mean FA skeleton, representing the centres of all tracts common to all participants; (v) to exclude the anatomical regions with lower probability belonging to the major WM tract area, the default threshold (FA = 0.2) was used to create the final WM skeleton and each aligned FA data were then projected onto this WM skeleton by ﬁlling the skeleton with FA values from the nearest relevant tract centre; (vi) to further spatially transform the MD maps into the standard MNI space, the corresponding nonlinear warps and skeleton projection information were also applied to the MD map for each individual; and (vii) finally, the resultant skeletonised FA and MD maps served as inputs for the following voxel-wise statistical analyses.

### Statistical analysis

*(1) Analysis of the demographic and clinical data*. Analyses were performed using SPSS (version 22.0, IBM, Armonk, NY, USA). All data are presented as mean (standard deviation [SD]) for continuous variables and number (percentage) for discrete variables. Group comparisons were made using the nonparametric Kruskal–Wallis test with *post-hoc* analyses. When appropriate, chi-square (χ^2^) or Fisher’s exact tests were performed for categorical variables. Multivariate linear regression analyses were used to test the association between SVD type and the CHS (adjusted for age and sex) and MMSE (adjusted for sex, age and education) scores, respectively. *(2) Analysis of voxel-wise GMV and WM microstructural integrity.* The permutation analysis of the linear models (part of FSL)^30^ was used to conduct permutation-based nonparametric statistical inferences for investigating the regional GMV, FA and MD alterations between the four SVD subtypes and controls. More specifically, the ANCOVA with age, sex and TIV as nuisance variables was used for both the VBM and TBSS analyses. The null distribution of each possible statistical contrast was built from 10 000 random permutations. Within this design, the effect of the main factor ‘group’ (*F*-test) was first evaluated. Subsequently, a series of *post-hoc t*-tests for each relevant statistical contrast between the SVD subtypes and controls were performed to determine the group-specific alterations of regional GMV and WM microstructural integrity. For all voxel-wise image-based analyses, the threshold of statistical significance was set at a cluster-level *P *<* *0.05, with FWE corrected for multiple comparisons (after an initial cluster-forming threshold of *t *>* *3.1). Finally, we uploaded all un-thresholded voxel-wise statistical maps to the NeuroVault website via the following permanent link (https://neurovault.org/collections/8461/) for transparency and reusability of all statistical results from the VBM and TBSS analyses. *(3) Common and distinct patterns of the neuroanatomic alterations between the SVD subtypes*. To elucidate the similarity and heterogeneity of the neuroanatomic alterations between the SVD subtypes, a hierarchical clustering analysis for the voxel-wise GM/FA/MD alterations of the four SVD subtypes were conducted using Matlab R2016a (The Mathworks, Inc., Natick, MA, USA). For each image marker, the voxel-wise un-thresholded t-statistic maps of the direct group comparisons between the SVD subtypes and controls were first transformed into a vector representing the spatial pattern of neuroanatomical alterations in each SVD subgroup. Subsequently, Pearson correlation coefficients between each vector were calculated and further transformed into a distance metric (1-*r*) for the following hierarchical clustering analyses. Pairs of patterns that were close in proximity based on the linkage function by the average distance were placed into binary clusters until all samples were combined into a single large cluster to form a hierarchical tree (dendrogram). Finally, the Calinski–Harabasz criterion was used to determine the optimal number of clusters.^31^*(4) Identifying trend level neuroanatomic alterations among SVD subtypes.* Whole-brain voxel-wise permutation-based nonparametric tests using ANCOVA (age, sex and TIV served as nuisance variables) were performed to evaluate whether there was a corresponding trend pattern in their neuroanatomic alterations. After model estimation, two additional statistical contrasts were used to detect the anatomical regions that demonstrated a significant ascending or descending trend level alteration in GMV and WM microstructural integrity. Ascending linear contrast (image-makers increasing with SVD subtypes) were defined as [–3 –1 +1 +3] across SVD types 1, 2 and 3, and their descending linear contrast was defined as [+3 +1 –1 –3]. To investigate the spatial distribution of the identified trend level changes in GMV and WM microstructural integrities, we parcellated the whole-brain into seven regions, including the bilateral frontal, parietal, limbic, subcortical, temporal, occipital lobe and cerebellum using WFU PickAtlas.^32^ The percentage of voxels that demonstrated statistically significant linear trend level changes across the SVD subtypes was also calculated and visualized in a pie chart for each sub-region.

### Data availability

We uploaded all un-thresholded voxel-wise statistical maps to the NeuroVault website via the following permanent link (https://neurovault.org/collections/8461/) for transparency and reusability of all statistical results from the VBM and TBSS analyses. Other data are available from the corresponding author on request.

## Results

### Demographics of the four stratified SVD groups

**Figure 2:**
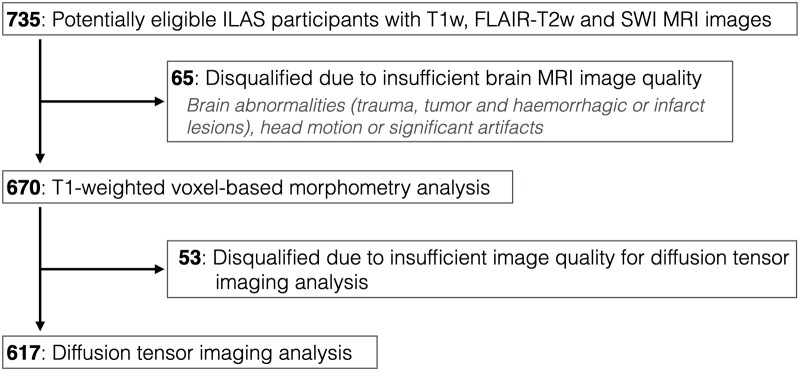
Study participants and the analysed neuroimaging data.

Among the 735 ILAS participants with eligible brain MRI images, 65 were excluded due to brain abnormalities (infarct, haemorrhage or tumor), head motion or missing data. Quality screening disqualified 53 candidates from subsequent diffusion tensor imaging analyses (670 for GMV analyses and 617 for WM microstructural analyses) (Fig. 2). There were two types of non-bleeding SVD (type 1: WMH without lacune; type 2: WMH with lacune) and two types of bleeding SVD (type 3: mixed CMB; type 4: strictly lobar CMB). Additional characteristics of the SVD markers in each classified SVD subtype are presented in Table 1. In bleeding SVD, particularly the type with mixed CMB, the presence of severe WMH was also prominent (81.5% and 55.9% of type 3 and type 4, respectively, had severe WMH; Table 1). Compared to the control group, the WMH volume ratios were significantly higher in each SVD group. Lacune were also present in the bleeding SVD; 42.6% and 11.8% of type 3 and type 4 had at least one lacune. There was no individual with isolated lacune in our population. Table 1 shows the demographics of each group. The Kruskal–Wallis nonparametric analyses showed that age and the presence of hypertension were significantly different among the five groups. *Post-hoc* analyses showed that each SVD group was older than the control group. Nevertheless, those with non-bleeding SVD and bleeding SVD with mixed CMB, e.g. SVD types 1, 2 and 3, but not bleeding SVD with strictly lobar CMB (type 4), had a higher prevalence of hypertension compared to the control group.

**Table 1:** Demographics and clinical/neuroimaging manifestations of the cerebral small vessel disease groups (*N* = 670)

	Control	SVD type 1	SVD type 2	SVD type 3	SVD type 4	*P*
	(*N *=* *312)	(*N *=* *230)	(*N *=* *40)	(*N *=* *54)	(*N *=* *34)	
Age, yr, mean (SD)	57.9 (5.9)	65.6 (8.1)^*^	67.8 (9.2)^*^	67.1 (10.3)^*^	64.3 (9.0)^*^	<0.001
Sex, male, *n* (%)	126 (40.4%)	111 (48.3%)	20 (50.0%)	26 (48.1%)	14 (41.2%)	0.163
Hypertension, *n* (%)	72 (23.1%)	98 (42.6%)	21 (52.5%)	22 (40.7%)	8 (23.5%)	<0.001
Education, yr, mean (SD)	8.7 (4.6)	5.7 (5.2)^*^	4.9 (4.3)^*^	5.6 (5.4)^*^	7.4 (5.9)	<0.001
Physical frailty status						
CHS score, mean (SD)	0.3 (0.6)	0.6 (0.8)^*^	(1.1)^*^	(1.1)^*^	0.6 (0.8)	<0.001
Physical frailty, *n* (%)	28 (0.9%)	8 (3.5%)	5 (12.5%)	6 (11.1%)	1 (2.9%)	<0.001
Cognitive function						
MMSE, mean (SD)	27.4 (2.6)	25.6 (3.3)^*^	24.6 (5.1)^*^	25.0 (4.7)^*^	25.6 (3.9)^*^	<0.001
10 min CVVLT	7.1 (1.6)	6.2 (2.1)^*^	5.8 (2.4)^*^	5.7 (2.2)^*^	6.0 (2.0)^*^	<0.001
Taylor Complex Figure Test	32.8 (4.2)	29.5 (7.0)^*^	28.5 (8.7)^*^	28.9 (9.1)^*^	29.3 (7.0)^*^	<0.001
Clock Drawing Test	8.5 (1.8)	7.5 (2.4)^*^	7.0 (3.0)^*^	7.2 (2.6)^*^	7.2 (2.7)^*^	<0.001
Global cognitive impairment, *n* (%)	10 (3.2%)	4 (1.7%)	2 (5.0%)	3 (5.6%)	3 (8.8%)	<0.001
SVD MRI markers						
Cerebral microbleed, present, *n* (%)	0	0	0	54 (100.0%)	34 (100.0%)	–
Severe WMH, present, *n* (%)	0	230 (100.0%)	40 (100.0%)	44 (81.5%)	19 (55.9%)	–
WMH volume ratio, 10^–3^	0.3 (0.2)	2.5 (3.0)^*^	4.1 (4.4)^*^	4.5 (4.8)^*^	2.8 (3.9)^*^^†^	<0.001
Lacune, present, *n* (%)	0	0	40 (100.0%)	23 (42.6%)	4 (11.8%)	–

CHS, Cardiosvascular Health Study; CVVLT, Chinese version Verbal Learning Test; MMSE, Mini-Mental State Examination; SVD, cerebral small vessel disease; WMH, white-matter hyperintensities.

* Significantly different from the control group in the *post hoc* analyses.

† Significantly different from SVD type 2 in the *post hoc* analyses.

### Physical and cognitive performances in SVD subtypes

The CHS and neuropsychological scores were significantly different among the five groups (Table 1). The non-bleeding SVD (types 1 and 2) and bleeding SVD with mixed CMBs (type 3) groups had similar clinical characteristics. The three groups had higher CHS and lower neuropsychological scores, yet lower education years compared to the control group. The group with bleeding SVD with strictly lobar CMB (type 4) had significantly lower MMSE and other neuropsychological scores than the control group despite having similar education years and CHS scores. The prevalence of physical frailty and global cognitive impairment were also different among the five groups, with both being higher in the SVD groups. Physical frailty was most prevalent in SVD type 2 and type 3 groups while the SVD type 4 group had the highest prevalence of global cognitive impairment. To adjust for confounders associated with physical frailty and cognitive function, we performed multivariate linear regression analyses. Since the SVD type 4 group had a similar CHS score to the control group, we did not include them into analyses for physical frail status. The results showed that the SVD types 1, 2 and 3 had significantly higher CHS scores than the control groups, which were independent of age and sex (Model 1 in Table 2). Linear regression analyses indicated an association between the SVD groups and CHS score, e.g. control < SVD type 1 < type 2 < type 3. Poorer cognitive functions were also significantly associated with the SVD groups, which was independent of age, sex and education (Model 1 in Table 2). The trend association between the SVD groups and all neuropsychological scores—control > SVD type 1 > type 2 > type 3 > type 4—in the multivariate linear regression analyses confirmed the significance of lower cognitive function in the SVD type 4 group.

**Table 2:** Multivariate linear regression analysis results of the physical frailty and cognitive function assessment scores

	*B*	95% CI	*P*
Physical frailty: Cardiovascular Health Study score (age and sex-adjusted)			
Model 1: SVD groups as linear variables (1 = control, 2 = SVD type 1, 3 = SVD type 2, 4 = SVD type 3)	0.10	0.04–0.16	0.002
Model 2: SVD groups as class variables, versus control respectively			
SVD type 1	0.02	−0.11 to 0.14	0.802
SVD type 2	0.35	0.14–0.56	0.001
SVD type 3	0.38	0.18–0.58	<0.001
Cognitive function: Mini-Mental State Examination score (age, sex and education-adjusted)			
Model 1: SVD groups as linear variables (1 = control, 2 = SVD type 1, 3 = SVD type 2, 4 = SVD type 3, 5= SVD type 4)	−0.20	−0.39 to −0.02	0.028
Model 2: SVD groups as class variable, versus control respectively			
SVD type 1	−0.28	−0.73 to 0.18	0.230
SVD type 2	−0.21	−0.62 to 0.20	0.311
SVD type 3	−0.55	−1.31 to 0.22	0.159
SVD type 4	−0.70	−1.52 to 0.11	0.091
Cognitive function: 10 min Chinese version Verbal Learning Test (age, sex and education-adjusted)			
Model 1: SVD groups as linear variables (1 = control, 2 = SVD type 1, 3 = SVD type 2, 4 = SVD type 3, 5= SVD type 4)	−0.18	−0.30 to −0.05	0.005
Model 2: SVD groups as class variable, versus control respectively			
SVD type 1	−0.46	−0.80 to −0.12	0.007
SVD type 2	−0.18	−0.46 to 0.09	0.181
SVD type 3	−0.52	−0.99 to −0.05	0.031
SVD type 4	−0.65	−1.20 to −0.10	0.020
Cognitive function: Taylor Complex Figure Test (age, sex and education-adjusted)			
Model 1: SVD groups as linear variables (1 = control, 2 = SVD type 1, 3 = SVD type 2, 4 = SVD type 3, 5 = SVD type 4)	−0.41	−0.79 to −0.04	0.030
Model 2: SVD groups as class variable, versus control respectively			
SVD type 1	−2.15	−3.55 to −0.76	0.003
SVD type 2	0.03	−1.25 to 1.31	0.961
SVD type 3	−1.97	−4.24 to 0.31	0.090
SVD type 4	−3.46	−6.17 to −0.75	0.013
Cognitive function: Clock Drawing Test (age, sex and education-adjusted)			
Model 1: SVD groups as linear variables (1 = control, 2 = SVD type 1, 3 = SVD type 2, 4 = SVD type 3, 5 = SVD type 4)	−0.15	−0.28 to −0.02	0.024
Model 2: SVD groups as class variable, versus control respectively			
SVD type 1	−0.15	−0.50 to 0.20	0.404
SVD type 2	−0.18	−0.48 to 0.12	0.246
SVD type 3	−0.32	−0.86 to 0.22	0.244
SVD type 4	−0.80	−1.43 to −0.18	0.012

SVD, cerebral small vessel disease.

We than analysed the physical and cognitive functions in each SVD type relative to control group with age, sex or education adjustment (Model 2 in Table 2). In the analyses for physical frailty, the results showed that SVD type 2 and type 3 groups had significantly higher CHS scores than controls. As for the cognitive functions, there were individual cognitive characteristics in each SVD type. The results showed that relative to controls, SVD type 1 had poorer verbal memory and visuospatial functions, SVD type 3 had poorer verbal memory and SVD type 4 had poorer performances in all cognitive domains (verbal memory, visuospatial and executive functions).

### Neuroanatomic alterations: macrostructural GMV analyses

**Figure 3:**
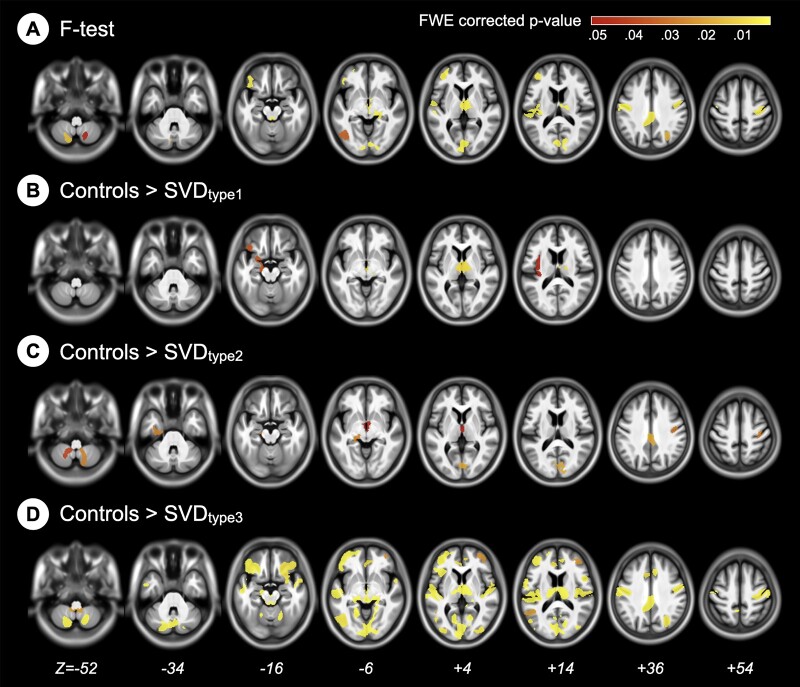
**Regional grey-matter volume comparisons among the groups**. The hot colour maps show the cluster-level statistics with the FWE-corrected *P* of the GMV. Statistically significant GMV differences between groups are highlighted in yellow or red. GMV, grey-matter volume; SVD, cerebral small vessel disease.

The results showed that the regions with GMV differences among groups were distributed bilaterally and diffusely from all cerebral lobes to the cerebellum and from the subcortical to the cortical grey matter in SVD groups (Fig. 3a). *Post-hoc* analyses further showed that the group differences were contributed by the differences between the control group and the SVD types 1, 2 and 3 groups, but not the type 4 group (Fig. 3b and c). The SVD types 1, 2 and 3 groups had lower GMVs in several brain regions compared to the control group (Supplementary Table 1). There was no significant difference in regional GMVs between the control and the SVD type 4 groups.

### Neuroanatomic alterations: microstructural WM integrity analyses

**Figure 4:**
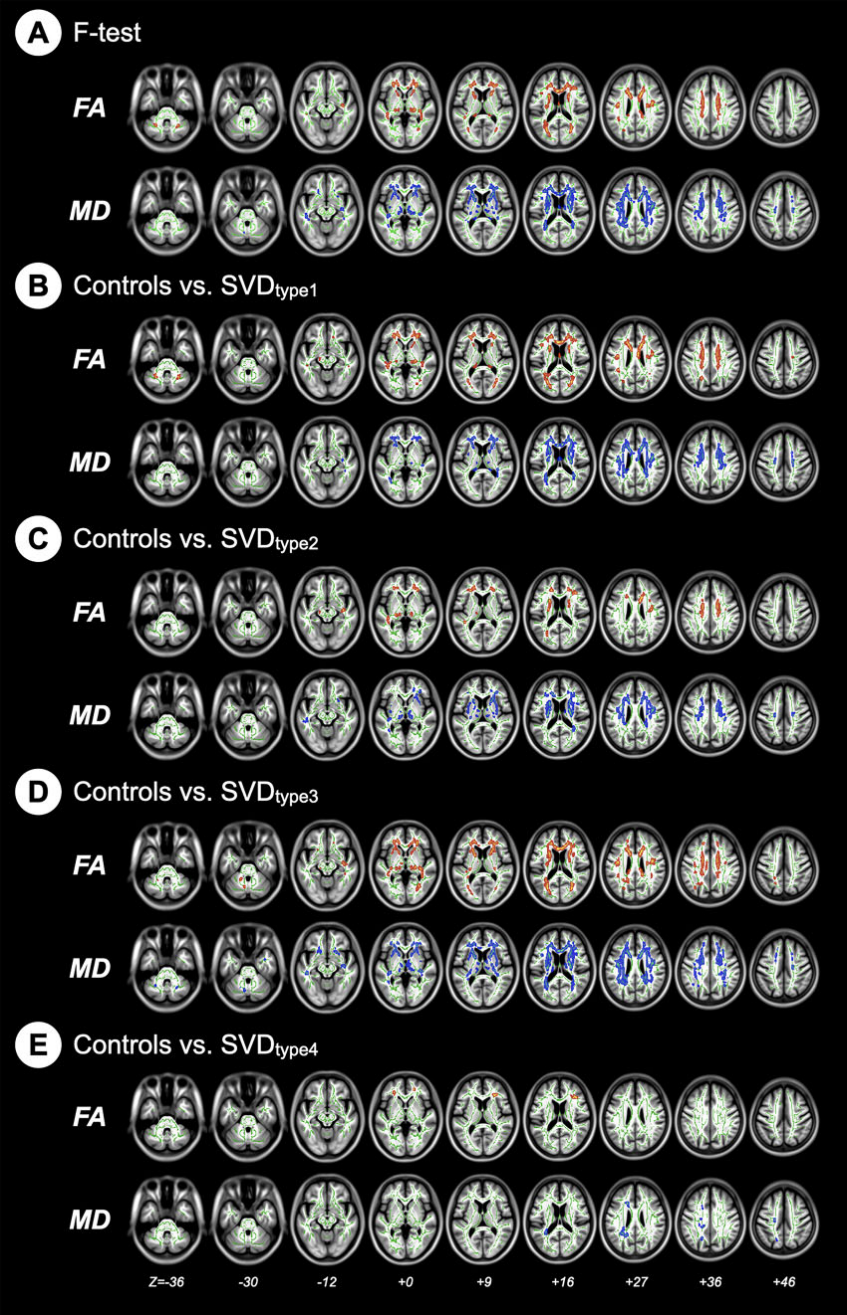
**Comparison of the white-matter microstructure measurements among the groups**. Diffusion indices between groups that are statistically significant are highlighted in red for FA and blue for MD. FA, fractional anisotropy; MD, mean diffusivity; SVD, cerebral small vessel disease.

There were also significant group differences in both FA and MD values, particularly over the frontal and subcortical regions (Fig. 4a). The *post-hoc* analyses showed that each SVD type had significantly lower regional FA and higher regional MD relative to the control group (Fig. 4b–e; Supplementary Tables 2 and 3). Notably, in contrast to the GMV analyses, which showed no significant volumetric alteration in GM, the SVD type 4 group had significantly abnormal WM microstructure integrity compared to the control group (Fig. 4e;Supplementary Tables 2 and 3).

### SVD type 4 has a distinct pattern of neuroanatomic alterations

**Figure 5:**
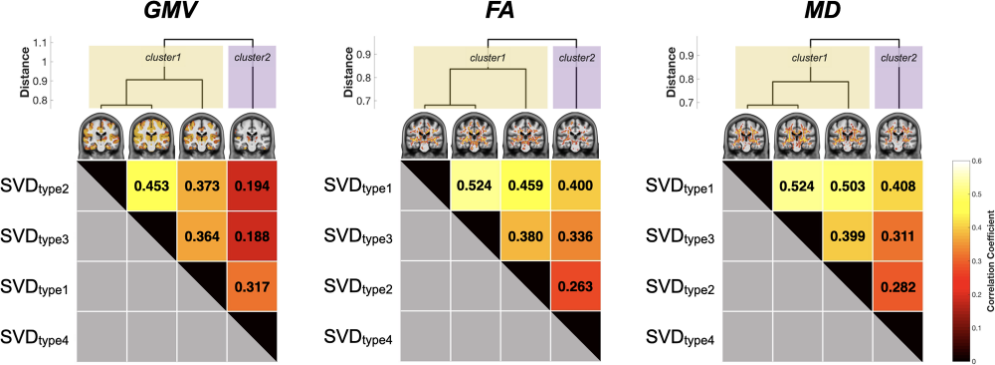
**Dendrogram of similarity in neuroanatomic changes among subtypes of cerebral small vessel disease**. The four statistical patterns of GMV, FA and MD images were grouped into two distinct clusters using a hierarchical clustering analysis. FA, fractional anisotropy; GMV, grey-matter volume; MD, mean diffusivity; SVD, cerebral small vessel disease; WM, white matter.

To elucidate the similarity and heterogeneity of neuroanatomic alterations among the SVD subtypes, a hierarchical clustering analysis of the spatial pattern of the GMV and WM microstructural (FA/MD measures) alterations of the four SVD subtypes was conducted. This demonstrated that SVD type 4 had neuroanatomic alterations distinct from the other three SVD types in both GMV and WM microstructural measurements (Fig. 5). SVD types 1, 2 and 3 were grouped as the same subset with more neuroanatomic similarities, whereas SVD types 2 and 3 had the most similar regional GMV changes and SVD types 1 and 3 had the most similar WM microstructural changes (Fig. 5).

### SVD types 1, 2 and 3 had similar neuroanatomic alteration patterns but different severity

**Figure 6:**
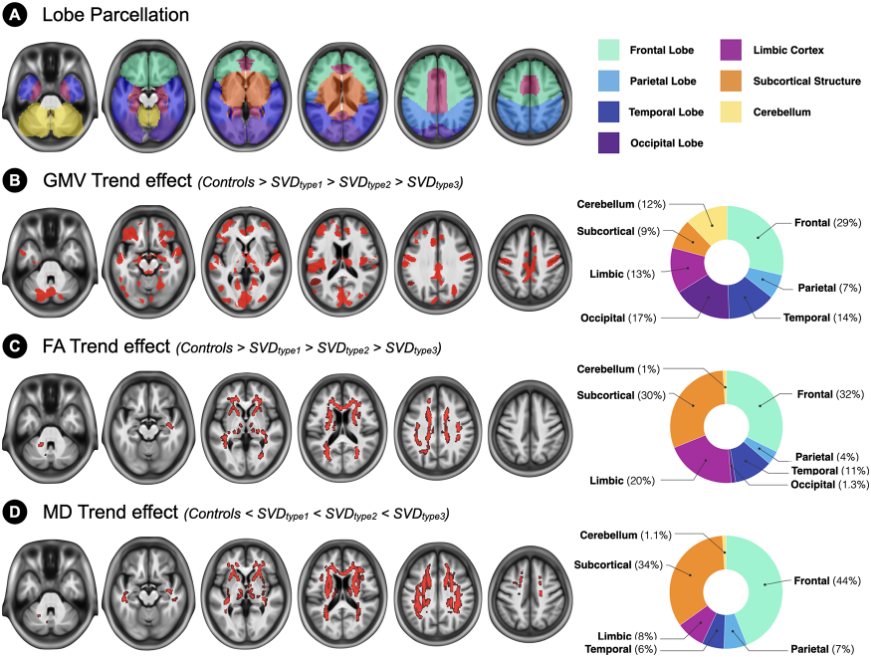
**Trend pattern and the regional distributions of neuroanatomic alterations among the subtypes of cerebral small vessel disease**. **(A)** Parcellation of the whole-brain region into seven cortical-subcortical-cerebellum subdivisions. **(B)** Regions with a statistically significant trend of group differences in GMV are shown in red. **(C)** Regions with a statistically significant trend of group differences in FA are shown in red. **(D)** Regions with a statistically significant trend of group differences in MD are shown in red. The pie charts on the right side demonstrate the geographic distributions of the identified neuroanatomic alternations in cerebral SVD types 1, 2 and 3. FA, fractional anisotropy; GMV, grey-matter volume; MD, mean diffusivity; SVD, cerebral small vessel disease.

Because of the trend of severity and prevalence in physical frailty and cognitive impairment among SVD types 1, 2 and 3 groups (e.g. SVD type 3 > type 2 > type 1; Table 2), we tested whether there was a corresponding trend pattern in their neuroanatomic alterations. The results of the voxel-wise trend level analyses indicated that there was a trend pattern in the GMV and WM microstructural changes among SVD types 1, 2 and 3. SVD type 3 had the most severe GM atrophy and WM integrity impairment among the three SVD subtypes (Fig. 6). To investigate the spatial distribution of the identified trend level association between the SVD subtypes and neuroanatomic changes, we calculated the ratio of the regional distributions of GMV and FA/MD changes relative to the control group with the seven pre-identified cortical-subcortical-cerebellum subdivisions (Fig. 6; Supplementary Tables 1–3). The results showed that the distribution of GMV reduction was diffuse, involving all cerebral lobes and the cerebellum, with the largest clusters over the frontal cortex followed by the occipital cortex, in the SVD types 1, 2 and 3 groups. By contrast, the distribution of disintegrated WM microstructures in the three SVD groups was primarily over the frontal and subcortical regions.

## Discussion

This study evaluated the validity of the proposed MRI marker-based classification method for SVD by testing whether stratified SVD subtypes had distinct clinical features and neuroanatomic changes. We found that SVD type 4 had distinct clinical manifestations and neuroanatomic alterations, including the GMV and WM microstructure. SVD types 1, 2 and 3 had similar patterns but different severity of neuroanatomic and clinical manifestations.

Our stratification scheme considers three common and easily assessed SVD markers. The differentiation between bleeding and non-bleeding SVD is achieved in the first step of the stratification scheme. We further stratified bleeding SVDs into different subtypes with CMBs of specific geographic features that have distinct underlying microvasculopathy, for example, strictly lobar CMBs as CAA and mixed CMBs as arteriosclerosis/lipohyalinosis microvasculopathy.^9^^,^^10^^,^^21^^,^^33–36^ Regarding the non-bleeding SVDs, subjects with severe WMHs were further divided depending on whether a lacune was present. Previous studies that studied the significance or effects of SVD have only focussed on individual SVD markers, mostly the WMH,^37^^,^^38^ and contradicted the actual circumstance that patients with SVD usually have several coexisted SVD markers in the brain. The latest proposed SVD measurement, the SVD score, includes more SVD markers. However, it only counts the number of SVD markers without considering their natures.^6–8^ Therefore, this classification method might reflect the severity, but not the pathogenesis, of SVD. Our proposed MRI-based SVD phenotyping method tries to address these concerns and could be used by clinicians to interpret the neuroimaging findings in SVD patients and by researchers to study the pathophysiology of SVD with specific pathogenesis.

Several studies have revealed that CMBs of strictly lobar regions have clinical associations distinct from CMBs of other distribution patterns.^33^ Subjects with mixed CMBs are more likely to have hypertension, elevated blood pressure and other features of hypertensive microvasculopathy.^33^ Those with strict lobar CMB are associated with features of CAA such as APOEɛ4 genotype and amyloid accumulation on brain positron emission tomography.^9^^,^^10^^,^^21^^,^^33–36^ Our finding of distinct clinical and neuroimaging features in SVD type 4 (strictly lobar CMBs) is consistent with those observations. The SVD type 4 group had less prevalence of hypertension and more prominent cognitive impairment, but preserved physical function, compared to the other SVD types. Their neuroanatomic changes also differed from the other SVD types. SVD type 4 had preserved regional GMVs but abnormal WM microstructures and poorer cognition. Previous studies have reported cortical atrophy in CAA patients who were older or with a history of stroke (intracerebral haemorrhage) or dementia.^39–41^ Our study population consisted of younger, community-based, non-stroke, non-demented, functionally preserved individuals. Therefore, our results might reflect an earlier or prodromal stage of disease when brain atrophy has not yet developed. The WM microstructures, where the cerebral microvessels are located, however, may alter before remote cortical changes occur. Our postulation is in line with the current consensus about the pathophysiology of SVD, which supports GM atrophy as a secondary change to WM injury during the disease process.^37^

Subjects with SVD types 1, 2 and 3 were more frequently to have hypertension and be less educated which results imply these SVD subtypes as hypertensive microvasculoapthy, e.g. arteriosclerosis/lipohyalinosis. The present study showing a significant association between physical frailty and SVD types 1, 2 and 3 suggests that arteriosclerosis/lipohyalinosis SVD might be more strongly correlated with physical frailty compared to CAA SVD, during the early or preclinical stage.

Our stratification method may also be used to evaluate non-bleeding and bleeding categories of arteriosclerosis/lipohyalinosis SVD. The present study showed that SVDs with mixed CMBs (SVD type 3) had more profound clinical and neuroanatomic abnormalities than severe WMH alone (SVD type 1) and severe WMH combined with lacune(s) (SVD type 2). These results are in agreement with previous studies which showed that CMB, particularly deep CMB, seldom exists as a single MRI marker but is always combined with other MRI markers.^6^^,^^42^ Therefore, the presence of CMB in SVD of arteriosclerosis/lipohyalinosis might represent a more advanced stage with more SVD burden. The present study also revealed the potential trajectories of GM and WM structural alterations in SVD of arteriosclerosis/lipohyalinosis. In SVD types 1, 2 and 3, the regions with GM atrophy were diffusely distributed and included all of the cerebral lobe and cerebellum, with the largest clusters over the frontal region followed by the occipital cortex. In contrast to the diffuse change of GMV, WM microstructure abnormalities in SVD types 1, 2 and 3 were mostly clustered over the frontal lobe and subcortical regions. These results reflect that the pathophysiology of SVD types 1, 2 and 3 might be initial frontal-subcortical circuit disruption followed by remote, diffuse cortical damage.

There are potential limitations of our proposed classification scheme. CAA and arteriosclerosis/lipohyalinosis can coexist in older patients, those with a history of stroke or dementia, or at a more advanced stage of SVD, and CMB distribution can extend to regions other than the lobar subcortical areas.^43^^,^^44^ Therefore, our stratification scheme using CMB locations to differentiate SVD phenotypes might be challenging in such a population. In addition, the present study did not consider the hereditary SVD, such as cerebral autosomal dominant arteriopathy with subcortical infarcts and leukoencephalopathy (CADASIL), and other rare aetiologies of SVD in our population.^43^^,^^45^^,^^46^ We assessed the severity of WMH using semiautomatic volumetric measurement since it offers a more reliable, sensitive, and objective alternative to visual rating scales.^47^ The present stratification scheme defined the presence of severe WMH as >50th percentile of WMH/TIV ratio in our study population. The cut-off point of the WMH volume ratio for defining severe WMH might differ between populations. Last but not the least, the present findings were from the community-based community who had no stroke or dementia. Whether the stratification method and yielded findings could be generalized to other population with evident clinical events needs more validation studies.

In conclusion, our MRI-based stratification scheme highlights the distinct features of SVD and possibly the underlying pathogenesis. This stratification scheme requires further testing with other ethnicities and symptomatic (dementia or/and stroke) patients and longitudinal studies to evaluate its prediction ability for disease prognosis.

## Supplementary material

Supplementary material is available at *Brain Communications* online.

## Supplementary Material

fcab107_Supplementary_Data

## Abbreviations

CAA =: cerebral amyloid angiopathy
CHS =: Cardiovascular Health Study
CMB =: cerebral microbleed
FA =: fractional anisotropy
GM =: grey matter
GMV =: grey-matter volume
ILAS =: I-Lan Longitudinal Aging Study
MD =: mean diffusivity
MMSE =: Mini-Mental State Examination
SPM =: Statistical Parametric Mapping
SVD =: cerebral small vessel disease
TBSS =: tract-based spatial statistics
TIV =: total intracranial volume
WM =: white matter
WMH =: white-matter hyperintensities
