# Supplementary material for: Classification differentiates clinical and neuroanatomic features of cerebral small vessel disease
Source: Brain Commun. 2021 May 20;3(2):fcab107. doi: 10.1093/braincomms/fcab107 (PMC8196251; doi:10.1093/braincomms/fcab107)
Supplement: fcab107_Supplementary_Data [file fcab107_supplementary_data.docx]

**Supplementary Table 1 Anatomical region with significant grey-matter volume changes**

| COG coordinates | | | Cluster size | Anatomical Region | | |
| --- | --- | --- | --- | --- | --- | --- |
| x | y | z |  | Side | Lobe | Region |
| **F-test** | | | | |  |  |
| -49 | -17 | 24 | 2845 | Left | Parietal Lobe | Postcentral Gyrus |
| 1 | -85 | 4 | 2390 | Right | Occipital Lobe | Lingual Gyrus |
| -38 | 41 | 1 | 2150 | Left | Frontal Lobe | Sub-Gyral |
| 7 | -15 | 1 | 2058 | Right | Sub-lobar | Thalamus |
| -1 | -34 | 38 | 2038 | Left | Limbic Lobe | Cingulate Gyrus |
| 43 | -14 | 45 | 1803 | Right | Frontal Lobe | Precentral Gyrus |
| -14 | -75 | -46 | 1138 | Left | Cerebellum | - |
| 31 | -72 | 29 | 1040 | Right | Occipital Lobe | Sub-Gyral |
| -45 | -67 | -8 | 706 | Left | Occipital Lobe | Sub-Gyral |
| 11 | -31 | 71 | 689 | Right | Parietal Lobe | Paracentral Lobule |
| 16 | -70 | -49 | 595 | Right | Cerebellum | - |
| **Controls > SVD_type1_** | | | | |  |  |
| 4 | -11 | 4 | 1788 | Right | Sub-lobar | Thalamus |
| -28 | 9 | -18 | 805 | Left | Frontal Lobe | Sub-Gyral |
| -36 | -8 | 15 | 685 | Left | Sub-lobar | Insula |
| **Controls > SVD_type2_** | | | | |  |  |
| 1 | -32 | 36 | 1201 | Right | Limbic Lobe | Posterior Cingulate |
| 6 | -82 | 12 | 1166 | Right | Occipital Lobe | Cuneus |
| -28 | -21 | -26 | 950 | Left | Limbic Lobe | Parahippocampal Gyrus |
| 14 | -64 | -52 | 912 | Right | Cerebellum | - |
| 41 | -16 | 44 | 885 | Right | Frontal Lobe | Sub-Gyral |
| -17 | -54 | -54 | 707 | Left | Cerebellum | - |
| 2 | -7 | -1 | 634 | Right | Sub-lobar | Third Ventricle |
| **Controls > SVD_type3_** | | | | |  |  |
| 1 | -52 | 0 | 12296 | Left | Cerebellum | Culmen |
| -52 | -16 | 14 | 8229 | Left | Parietal Lobe | Postcentral Gyrus |
| -36 | 41 | -1 | 6898 | Left | Frontal Lobe | Sub-Gyral |
| -5 | -74 | -42 | 5111 | Left | Cerebellum | - |
| 1 | -21 | 38 | 4206 | Right | Limbic Lobe | Posterior Cingulate |
| 45 | -13 | 47 | 3090 | Right | Frontal Lobe | Postcentral Gyrus |
| 50 | -4 | 6 | 2954 | Right | Sub-lobar | Insula |
| 31 | -77 | 25 | 2384 | Right | Occipital Lobe | Middle Occipital Gyrus |
| -44 | -72 | -3 | 2183 | Left | Occipital Lobe | Middle Occipital Gyrus |
| 31 | 29 | -16 | 2138 | Right | Frontal Lobe | Inferior Frontal Gyrus |
| -3 | 36 | 12 | 1705 | Left | Limbic Lobe | Anterior Cingulate |
| -51 | -54 | 19 | 989 | Left | Temporal Lobe | Superior Temporal Gyrus |
| 2 | -48 | -55 | 970 | Right | Cerebellum | - |
| 38 | 49 | 6 | 914 | Right | Frontal Lobe | Middle Frontal Gyrus |
| 10 | -29 | 72 | 749 | Right | Frontal Lobe | Paracentral Lobule |
| **Trend effect (Controls> SVD_type1_ >SVD_type2_ >SVD_type3_)** | | | | | | |
| 6 | -57 | 3 | 19946 | Right | Occipital Lobe | Lingual Gyrus |
| 1 | -5 | 35 | 10060 | Right | Limbic Lobe | Anterior Cingulate |
| -51 | -15 | 11 | 9851 | Left | Frontal Lobe | Precentral Gyrus |
| -4 | -70 | -45 | 9206 | Left | Cerebellum | Inferior Semi-Lunar Lobule |
| 48 | -11 | 23 | 6895 | Right | Sub-lobar | Extra-Nuclear |
| -35 | 42 | 4 | 6866 | Left | Frontal Lobe | Sub-Gyral |
| -45 | -70 | -3 | 3097 | Left | Occipital Lobe | Middle Occipital Gyrus |
| 34 | 39 | -5 | 2818 | Right | Frontal Lobe | Sub-Gyral |
| -49 | -54 | 20 | 1096 | Left | Temporal Lobe | Superior Temporal Gyrus |

**Supplementary Table 2 Anatomical regions with significant fractional anisotropy changes**

| COG coordinates | | | Cluster size | Anatomical Region | | |
| --- | --- | --- | --- | --- | --- | --- |
| x | y | z |  | Side | Lobe | Region |
| **F-test** | | | | |  |  |
| 4 | 16 | 19 | 5454 | Right | Limbic Lobe | Anterior Cingulate |
| -27 | -64 | 17 | 462 | Left | Temporal Lobe | Sub-Gyral |
| -20 | 9 | 12 | 416 | Left | Sub-lobar | Extra-Nuclear |
| 37 | -8 | 27 | 350 | Right | Frontal Lobe | Precentral Gyrus |
| -40 | -35 | 0 | 192 | Left | Temporal Lobe | Sub-Gyral |
| 18 | -22 | -2 | 188 | Right | Sub-lobar | Thalamus |
| 29 | -64 | 18 | 177 | Right | Limbic Lobe | Posterior Cingulate |
| -38 | -11 | 28 | 164 | Left | Frontal Lobe | Precentral Gyrus |
| 31 | -67 | 7 | 110 | Right | Occipital Lobe | Middle Occipital Gyrus |
| 39 | -34 | -2 | 97 | Right | Temporal Lobe | Sub-Gyral |
| -35 | 14 | 21 | 93 | Left | Frontal Lobe | Sub-Gyral |
| -37 | -42 | 30 | 91 | Left | Parietal Lobe | Inferior Parietal Lobule |
| 25 | -57 | -38 | 83 | Right | Cerebellum | Cerebellar Tonsil |
| -19 | -22 | -4 | 81 | Left | Sub-lobar | Extra-Nuclear |
| 29 | -11 | 24 | 79 | Right | Sub-lobar | Extra-Nuclear |
| 35 | -24 | -1 | 76 | Right | Sub-lobar | Extra-Nuclear |
| 38 | -11 | -13 | 72 | Right | Temporal Lobe | Sub-Gyral |
| 0 | -8 | 17 | 58 | Left | Sub-lobar | Extra-Nuclear |
| -25 | -56 | -37 | 55 | Left | Cerebellum | Cerebellar Tonsil |
| -13 | -22 | 3 | 48 | Left | Sub-lobar | Thalamus |
| -17 | -34 | 9 | 48 | Left | Sub-lobar | Extra-Nuclear |
| 10 | 28 | -4 | 45 | Right | Sub-lobar | Extra-Nuclear |
| -28 | -15 | 16 | 39 | Left | Sub-lobar | Claustrum |
| 17 | -34 | 9 | 36 | Right | Sub-lobar | Extra-Nuclear |
| -15 | -42 | 41 | 35 | Left | Frontal Lobe | Precuneus |
| **Controls > SVD_type1_** | | | | |  |  |
| 2 | 16 | 20 | 5410 | Left | Temporal Lobe | Sub-Gyral |
| -27 | -64 | 17 | 564 | Right | Limbic Lobe | Posterior Cingulate |
| 29 | -67 | 14 | 509 | Right | Sub-lobar | Extra-Nuclear |
| 21 | 11 | 12 | 268 | Right | Sub-lobar | Extra-Nuclear |
| 37 | -3 | 25 | 226 | Left | Temporal Lobe | Sub-Gyral |
| -40 | -31 | 0 | 199 | Right | Sub-lobar | Thalamus |
| 19 | -22 | -2 | 153 | Right | Cerebellum | Cerebellar Tonsil |
| 26 | -57 | -38 | 121 | Left | Frontal Lobe | Precentral Gyrus |
| -39 | -13 | 29 | 121 | Left | Brainstem | Midbrain |
| -18 | -19 | -6 | 119 | Left | Cerebellum | Cerebellar Tonsil |
| -26 | -55 | -38 | 114 | Left | Sub-lobar | Extra-Nuclear |
| -21 | 7 | 16 | 111 | Right | Temporal Lobe | Sub-Gyral |
| 48 | -37 | -7 | 110 | Right | Sub-lobar | Extra-Nuclear |
| 39 | -28 | -2 | 101 | Left | Parietal Lobe | Inferior Parietal Lobule |
| -37 | -42 | 29 | 96 | Left | Sub-lobar | Thalamus |
| -16 | -33 | 9 | 83 | Left | Sub-lobar | Extra-Nuclear |
| 1 | -9 | 17 | 78 | Left | Parietal Lobe | Precuneus |
| -19 | -62 | 37 | 74 | Left | Temporal Lobe | Sub-Gyral |
| -45 | -36 | -7 | 68 | Right | Sub-lobar | Extra-Nuclear |
| 29 | -11 | 24 | 55 | Left | Frontal Lobe | Sub-Gyral |
| -24 | 16 | 31 | 53 | Right | Sub-lobar | Extra-Nuclear |
| 18 | -34 | 9 | 52 | Left | Frontal Lobe | Sub-Gyral |
| -39 | 15 | 17 | 51 | Left | Sub-lobar | Extra-Nuclear |
| -19 | 18 | 4 | 46 | Left | Temporal Lobe | Sub-Gyral |
| **Controls > SVD_type2_** | | | | |  |  |
| 19 | 18 | 21 | 1126 | Right | Frontal Lobe | Sub-Gyral |
| -21 | 31 | 14 | 675 | Left | Frontal Lobe | Sub-Gyral |
| -17 | -12 | 36 | 289 | Left | Limbic Lobe | Cingulate Gyrus |
| 36 | -7 | 25 | 217 | Right | Frontal Lobe | Sub-Gyral |
| -22 | 2 | 17 | 174 | Left | Sub-lobar | Extra-Nuclear |
| -40 | -36 | 3 | 129 | Left | Temporal Lobe | Sub-Gyral |
| -20 | -21 | -4 | 98 | Left | Sub-lobar | Extra-Nuclear |
| 34 | 27 | 16 | 84 | Right | Frontal Lobe | Sub-Gyral |
| 37 | -14 | -11 | 83 | Right | Temporal Lobe | Sub-Gyral |
| 23 | 3 | 18 | 77 | Right | Sub-lobar | Extra-Nuclear |
| -28 | -66 | 18 | 63 | Left | Temporal Lobe | Sub-Gyral |
| -12 | -21 | 4 | 60 | Left | Sub-lobar | Thalamus |
| 13 | -21 | 2 | 53 | Right | Sub-lobar | Thalamus |
| 35 | 9 | 23 | 46 | Right | Frontal Lobe | Sub-Gyral |
| **Controls > SVD_type3_** | | | | |  |  |
| 1 | 14 | 17 | 7112 | * | * | Corpus Callosum |
| -27 | -62 | 17 | 646 | Left | Temporal Lobe | Sub-Gyral |
| -37 | -21 | 29 | 506 | Left | Frontal Lobe | Postcentral Gyrus |
| 37 | -7 | 26 | 467 | Right | Frontal Lobe | Precentral Gyrus |
| 36 | -19 | -5 | 372 | Right | Sub-lobar | Extra-Nuclear |
| 29 | -65 | 17 | 271 | Right | Limbic Lobe | Posterior Cingulate |
| 18 | -22 | -2 | 211 | Right | Sub-lobar | Thalamus |
| 39 | -35 | -4 | 173 | Right | Temporal Lobe | Sub-Gyral |
| 30 | -67 | 2 | 132 | Right | Occipital Lobe | Lingual Gyrus |
| -28 | -61 | 34 | 118 | Left | Parietal Lobe | Sub-Gyral |
| -38 | -34 | 3 | 106 | Left | Temporal Lobe | Sub-Gyral |
| -17 | -50 | 29 | 83 | Left | Limbic Lobe | Sub-Gyral |
| -19 | -22 | -4 | 78 | Left | Brainstem | Midbrain |
| 37 | -38 | 31 | 78 | Right | Parietal Lobe | Sub-Gyral |
| -30 | -25 | 5 | 69 | Left | Sub-lobar | Extra-Nuclear |
| -28 | -14 | 16 | 66 | Left | Sub-lobar | Claustrum |
| -8 | -18 | 35 | 64 | Left | Limbic Lobe | Cingulate Gyrus |
| -20 | -55 | 41 | 64 | Left | Parietal Lobe | Precuneus |
| -18 | -48 | -32 | 62 | Left | Cerebellum | - |
| -7 | -66 | -28 | 60 | Left | Cerebellum | Nodule |
| -13 | -22 | 1 | 58 | Left | Sub-lobar | Thalamus |
| -34 | 9 | 22 | 57 | Left | Frontal Lobe | Sub-Gyral |
| 17 | 34 | 35 | 53 | Right | Frontal Lobe | Medial Frontal Gyrus |
| -9 | -11 | 4 | 52 | Left | Sub-lobar | Thalamus |
| **Controls > SVD_type4_** | | | | |  |  |
| 31 | 31 | 12 | 181 | Right | Frontal Lobe | Sub-Gyral |
| -24 | 39 | -2 | 70 | Left | Frontal Lobe | Sub-Gyral |
| 19 | 45 | -2 | 51 | Right | Limbic Lobe | Anterior Cingulate |
| **Trend effect (Controls > SVD_type1_ > SVD_type2_ > SVD_type3_)** | | | | | | |
| 2 | 8 | 18 | 6675 | * | * | Corpus Callosum |
| -22 | 5 | 13 | 843 | Left | Sub-lobar | Extra-Nuclear |
| 37 | -6 | 26 | 584 | Right | Frontal Lobe | Sub-Gyral |
| 32 | -19 | -5 | 582 | Right | Sub-lobar | Lentiform Nucleus |
| -37 | -9 | 27 | 331 | Left | Frontal Lobe | Precentral Gyrus |
| -40 | -37 | 4 | 225 | Left | Temporal Lobe | Sub-Gyral |
| 39 | -36 | -3 | 217 | Right | Temporal Lobe | Sub-Gyral |
| -27 | 16 | -1 | 210 | Left | Sub-lobar | Extra-Nuclear |
| 28 | -63 | 17 | 190 | Right | Limbic Lobe | Posterior Cingulate |
| -37 | -39 | 31 | 135 | Left | Parietal Lobe | Inferior Parietal Lobule |
| -19 | -23 | -3 | 116 | Left | Brainstem | Midbrain |
| -25 | -60 | 33 | 107 | Left | Parietal Lobe | Sub-Gyral |
| 13 | -21 | -1 | 98 | Right | Sub-lobar | Thalamus |
| 29 | -65 | 1 | 87 | Right | Occipital Lobe | Lingual Gyrus |
| 38 | -36 | 31 | 76 | Right | Parietal Lobe | Sub-Gyral |
| -9 | -10 | 4 | 74 | Left | Sub-lobar | Thalamus |
| -13 | -22 | 1 | 70 | Left | Sub-lobar | Thalamus |
| -8 | -18 | 35 | 68 | Left | Limbic Lobe | Cingulate Gyrus |
| -7 | -66 | -28 | 60 | Left | Cerebellum | Nodule |
| -29 | -25 | 7 | 59 | Left | Sub-lobar | Extra-Nuclear |
| -43 | -31 | -8 | 56 | Left | Temporal Lobe | Sub-Gyral |
| 8 | -16 | 35 | 55 | Right | Limbic Lobe | Cingulate Gyrus |
| -37 | -49 | 2 | 54 | Left | Temporal Lobe | Sub-Gyral |
| -14 | -12 | 15 | 54 | Left | Sub-lobar | Thalamus |
| -34 | 9 | 22 | 54 | Left | Frontal Lobe | Sub-Gyral |
| -18 | -48 | -32 | 53 | Left | Cerebellum | - |
| 10 | -10 | 1 | 50 | Right | Sub-lobar | Thalamus |

**Supplementary Table 3 Anatomical regions with significant mean diffusivity changes**

| COG coordinates | | | Cluster size | Anatomical Region | | |
| --- | --- | --- | --- | --- | --- | --- |
| x | y | z |  | Side | Lobe | Region |
| **F-test** | | | | |  |  |
| 4 | 4 | 21 | 12748 | Right | Sub-lobar | Extra-Nuclear |
| -36 | -20 | 27 | 975 | Left | Frontal Lobe | Postcentral Gyrus |
| 12 | -16 | 5 | 359 | Right | Sub-lobar | Thalamus |
| 37 | -19 | -7 | 153 | Right | Sub-lobar | Extra-Nuclear |
| -12 | -21 | 4 | 122 | Left | Sub-lobar | Thalamus |
| -37 | -30 | 2 | 114 | Left | Sub-lobar | Extra-Nuclear |
| -9 | -10 | 5 | 90 | Left | Sub-lobar | Thalamus |
| -45 | -30 | -12 | 77 | Left | Temporal Lobe | Sub-Gyral |
| -38 | -46 | -6 | 74 | Left | Limbic Lobe | Parahippocampal Gyrus |
| 26 | -50 | 28 | 60 | Right | Parietal Lobe | Sub-Gyral |
| -7 | -66 | -26 | 49 | Left | Cerebellum | Nodule |
| -17 | -34 | 9 | 41 | Left | Sub-lobar | Thalamus |
| 0 | -9 | 17 | 40 | Left | Sub-lobar | Extra-Nuclear |
| -34 | -9 | 1 | 36 | Left | Sub-lobar | Extra-Nuclear |
| 35 | -34 | 9 | 35 | Right | Temporal Lobe | Sub-Gyral |
| 17 | -33 | 9 | 35 | Right | Sub-lobar | Extra-Nuclear |
| 3 | -8 | 13 | 35 | Right | Sub-lobar | Lateral Ventricle |
| -31 | -1 | 28 | 33 | Left | Frontal Lobe | Sub-Gyral |
| 41 | -39 | -9 | 30 | Right | Limbic Lobe | Parahippocampal Gyrus |
| 31 | -1 | 28 | 29 | Right | Frontal Lobe | Sub-Gyral |
| 30 | -63 | 18 | 27 | Right | Temporal Lobe | Sub-Gyral |
| **Controls < SVD_type1_** | | | | |  |  |
| 4 | 6 | 24 | 9139 | * | Sub-lobar | Extra-Nuclear |
| -36 | -15 | 28 | 382 | Left | Frontal Lobe | Postcentral Gyrus |
| 1 | -9 | 15 | 125 | Left | Sub-lobar | Extra-Nuclear |
| -37 | -42 | 30 | 124 | Left | Parietal Lobe | Inferior Parietal Lobule |
| -17 | -34 | 9 | 60 | Left | Sub-lobar | Extra-Nuclear |
| 33 | 22 | 18 | 60 | Right | Frontal Lobe | Sub-Gyral |
| -25 | -11 | 17 | 48 | Left | Sub-lobar | Extra-Nuclear |
| 35 | -34 | 9 | 44 | Right | Temporal Lobe | Sub-Gyral |
| -35 | -57 | -1 | 43 | Left | Temporal Lobe | Sub-Gyral |
| 13 | -19 | 7 | 43 | Right | Sub-lobar | Thalamus |
| 35 | -15 | -10 | 41 | Right | Sub-lobar | Lateral Ventricle |
| 18 | -34 | 9 | 40 | Right | Sub-lobar | Extra-Nuclear |
| 35 | -26 | 27 | 40 | Right | Frontal Lobe | Sub-Gyral |
| 39 | -24 | -3 | 39 | Right | Sub-lobar | Extra-Nuclear |
| -12 | -20 | 5 | 37 | Left | Sub-lobar | Thalamus |
| -29 | -65 | 19 | 36 | Left | Temporal Lobe | Sub-Gyral |
| 35 | -46 | 8 | 34 | Right | Sub-lobar | Extra-Nuclear |
| 30 | -4 | 17 | 33 | Right | Sub-lobar | Extra-Nuclear |
| 6 | -10 | 27 | 32 | Right | Limbic Lobe | Cingulate Gyrus |
| 40 | -40 | -9 | 29 | Right | Temporal Lobe | Sub-Gyral |
| 27 | -49 | 28 | 29 | Right | Frontal Lobe | Sub-Gyral |
| -27 | -6 | 39 | 29 | Left | Frontal Lobe | Sub-Gyral |
| **Controls < SVD_type2_** | | | | |  |  |
| -25 | -5 | 23 | 2006 | Left | Sub-lobar | Extra-Nuclear |
| 26 | 5 | 17 | 1982 | Right | Sub-lobar | Extra-Nuclear |
| 18 | 14 | 31 | 659 | Right | Limbic Lobe | Cingulate Gyrus |
| -17 | 21 | 26 | 523 | Left | Limbic Lobe | Cingulate Gyrus |
| 38 | 0 | 25 | 506 | Right | Frontal Lobe | Sub-Gyral |
| -36 | -10 | 26 | 251 | Left | Frontal Lobe | Precentral Gyrus |
| 33 | 34 | 8 | 206 | Right | Frontal Lobe | Sub-Gyral |
| 12 | -20 | 4 | 191 | Right | Sub-lobar | Thalamus |
| -12 | -21 | 3 | 132 | Left | Sub-lobar | Thalamus |
| 25 | -34 | 29 | 122 | Right | Frontal Lobe | Sub-Gyral |
| 11 | -9 | 6 | 114 | Right | Sub-lobar | Thalamus |
| 36 | -21 | 31 | 113 | Right | Frontal Lobe | Sub-Gyral |
| 31 | -46 | 18 | 107 | Right | Sub-lobar | Extra-Nuclear |
| 21 | -14 | 44 | 104 | Right | Limbic Lobe | Cingulate Gyrus |
| -48 | -30 | -11 | 84 | Left | Temporal Lobe | Sub-Gyral |
| 19 | 39 | 1 | 71 | Right | Frontal Lobe | Sub-Gyral |
| -28 | 29 | 16 | 70 | Left | Frontal Lobe | Sub-Gyral |
| -39 | -25 | -4 | 69 | Left | Sub-lobar | Claustrum |
| -38 | -48 | -5 | 54 | Left | Limbic Lobe | Sub-Gyral |
| -10 | -9 | 6 | 50 | Left | Sub-lobar | Thalamus |
| 53 | -37 | -6 | 49 | Right | Temporal Lobe | Sub-Gyral |
| **Controls < SVD_type3_** | | | | |  |  |
| -25 | 1 | 20 | 7174 | Left | Sub-lobar | Extra-Nuclear |
| 25 | 8 | 19 | 6405 | Right | Frontal Lobe | Sub-Gyral |
| 25 | -38 | 27 | 639 | Right | Sub-lobar | Extra-Nuclear |
| 36 | -22 | -3 | 291 | Right | Sub-lobar | Extra-Nuclear |
| -34 | -30 | 4 | 213 | Left | Sub-lobar | Extra-Nuclear |
| 12 | -10 | 9 | 168 | Right | Sub-lobar | Thalamus |
| -8 | -66 | -27 | 163 | Left | Cerebellum | Nodule |
| -13 | -22 | 9 | 162 | Left | Sub-lobar | Thalamus |
| 20 | -13 | 43 | 152 | Right | Limbic Lobe | Cingulate Gyrus |
| 11 | -20 | 2 | 109 | Right | Sub-lobar | Thalamus |
| -9 | -10 | 5 | 105 | Left | Sub-lobar | Thalamus |
| 10 | -10 | 1 | 95 | Right | Sub-lobar | Thalamus |
| -14 | 32 | 40 | 81 | Left | Frontal Lobe | Sub-Gyral |
| -46 | -29 | -12 | 80 | Left | Temporal Lobe | Sub-Gyral |
| 34 | 36 | 8 | 79 | Right | Frontal Lobe | Sub-Gyral |
| 42 | -3 | -24 | 66 | Right | Temporal Lobe | Sub-Gyral |
| 25 | -50 | 28 | 64 | Right | Parietal Lobe | Sub-Gyral |
| -29 | -61 | 18 | 58 | Left | Temporal Lobe | Sub-Gyral |
| 30 | -63 | 18 | 57 | Right | Temporal Lobe | Sub-Gyral |
| 30 | 11 | 38 | 57 | Right | Frontal Lobe | Sub-Gyral |
| 30 | -19 | -7 | 56 | Right | Sub-lobar | Extra-Nuclear |
| 23 | -59 | -38 | 55 | Right | Cerebellum | Cerebellar Tonsil |
| 31 | 24 | 32 | 53 | Right | Frontal Lobe | Sub-Gyral |
| 16 | 36 | 36 | 53 | Right | Frontal Lobe | Medial Frontal Gyrus |
| -30 | -60 | 32 | 50 | Left | Temporal Lobe | Sub-Gyral |
| 7 | -67 | -26 | 48 | Right | Cerebellum | Nodule |
| -24 | -58 | -39 | 46 | Left | Cerebellum | Cerebellar Tonsil |
| 5 | -9 | -1 | 41 | Right | Sub-lobar | Thalamus |
| -37 | -50 | -2 | 40 | Left | Temporal Lobe | Sub-Gyral |
| **Controls < SVD_type4_** | | | | |  |  |
| -26 | -48 | 23 | 183 | Left | Sub-lobar | Extra-Nuclear |
| -17 | 16 | 30 | 142 | Left | Frontal Lobe | Sub-Gyral |
| -23 | -34 | 31 | 118 | Left | Frontal Lobe | Sub-Gyral |
| -30 | -45 | 29 | 82 | Left | Parietal Lobe | Sub-Gyral |
| -17 | -62 | 39 | 64 | Left | Parietal Lobe | Precuneus |
| -20 | -17 | 44 | 56 | Left | Limbic Lobe | Cingulate Gyrus |
| **Trend effect (Controls < SVD_type1_ < SVD_type2_ < SVD_type3_)** | | | | | | |
| -25 | 0 | 20 | 8345 | Left | Sub-lobar | Extra-Nuclear |
| 25 | 7 | 18 | 7812 | Right | Sub-lobar | Extra-Nuclear |
| 25 | -38 | 26 | 637 | Right | Sub-lobar | Extra-Nuclear |
| -43 | -31 | -11 | 412 | Left | Temporal Lobe | Sub-Gyral |
| 35 | -18 | -7 | 358 | Right | Sub-lobar | Extra-Nuclear |
| -13 | -22 | 7 | 225 | Left | Sub-lobar | Thalamus |
| 42 | -4 | -25 | 137 | Right | Temporal Lobe | Sub-Gyral |
| -9 | -67 | -27 | 131 | Left | Cerebellum | Nodule |
| -9 | -10 | 4 | 130 | Left | Sub-lobar | Thalamus |
| 16 | 36 | 36 | 87 | Right | Frontal Lobe | Medial Frontal Gyrus |
| 37 | -33 | 6 | 72 | Right | Temporal Lobe | Sub-Gyral |
| 30 | 12 | 38 | 72 | Right | Frontal Lobe | Sub-Gyral |
| -25 | -58 | -38 | 68 | Left | Cerebellum | Cerebellar Tonsil |
| 25 | -50 | 29 | 63 | Right | Parietal Lobe | Sub-Gyral |
| 50 | -28 | -13 | 57 | Right | Temporal Lobe | Sub-Gyral |
| 5 | -10 | -1 | 55 | Right | Sub-lobar | Thalamus |
| 30 | -61 | 18 | 54 | Right | Temporal Lobe | Sub-Gyral |
| -29 | -61 | 18 | 54 | Left | Temporal Lobe | Sub-Gyral |
| -11 | -9 | 14 | 49 | Left | Sub-lobar | Thalamus |
| -49 | -10 | -22 | 47 | Left | Temporal Lobe | Sub-Gyral |
| -29 | 33 | 22 | 46 | Left | Frontal Lobe | Sub-Gyral |
| 30 | 22 | 34 | 45 | Right | Frontal Lobe | Sub-Gyral |
| -27 | 5 | 44 | 45 | Left | Frontal Lobe | Middle Frontal Gyrus |
